# ‘I Can Step outside My Comfort Zone.’

**DOI:** 10.3390/pharmacy5040059

**Published:** 2017-10-22

**Authors:** Morag C. E. McFadyen, Lesley Diack

**Affiliations:** School of Pharmacy and Life sciences, Robert Gordon University, Riverside East, Garthdee Road, Aberdeen AB10 7GJ, UK; h.l.diack@rgu.ac.uk

**Keywords:** evidence-based medicine, undergraduate pharmacy, thematic analysis, science practice integration, systematic review

## Abstract

On embarking upon such a multifactorial, professional degree as Pharmacy, students often find it difficult to meld the scientific- and practice-based components of the course. In final year of the undergraduate Masters of Pharmacy degree (MPharm) within the School of Pharmacy and Life Sciences at Robert Gordon University (RGU), students undertake a research project within a specific area. The aims of this study were to explore the effectiveness of a novel practice based approach to a biomedical science project, to identify elements of difficulty in the process, and to explore students’ perceptions and reflections. Final year students were assigned to perform a systematic literature review working within a defined area of pharmacovigilance. Students were given individual ownership of the research question and were able to choose a topic of interest. Following the successful completion of the assignment, students were invited to explore their attitudes and views of the project and reflect on the process through a focus group using a talking wall method. The findings clearly identified a shift in mindset from predominantly negative opinions initially to an overwhelming positive viewpoint.

## 1. Introduction: Evidence Based Medicine and Pharmacy

This paper investigates the views and attitudes of final year pharmacy students to the substitution of a science based project to a systematic review of a science topic. The innovative project was developed to highlight the centrality of the pharmacist’s role in evidence based practice and pharmacovigilance (otherwise known as ‘drug safety’, which involves monitoring adverse events associated with the use of pharmaceutical products). The responsibility for pharmacovigilance falls under the Medicines and Healthcare products Regulatory Agency (MHRA) [1]. MPharm students at RGU are introduced to the MHRA in the first year of the course. Within the United Kingdom (UK), the MHRA uses adverse drug event (ADE) data collected from patients and healthcare professionals such as the pharmacist (the ‘yellow card scheme’) in addition to clinical studies [2]. Using this information, the MHRA assesses whether the potential for benefit outweighs the potential for harm, which in turn leads to a decision being made on whether a particular product will be given a marketing authorization, knowledge and understanding of this process is vital for pharmacy students. Using a systematic approach to review scientific literature in their final year has highlighted this and how their understanding could be important in their professional practice.

**Figure 1:**
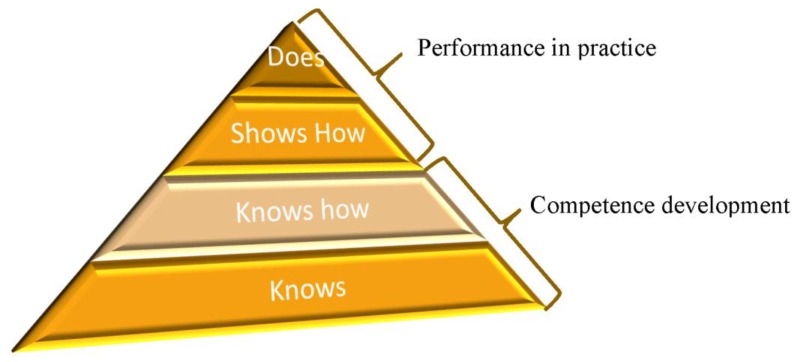
Miller’s Triangle [3].

In the UK, the General Pharmaceutical Council (GPhC) is the accrediting body for Pharmacy education in Great Britain and the statutory regulator for pharmacists. A GPhC-accredited Master of Pharmacy (MPharm) degree is part of the pathway from a first year MPharm student to registration as a pharmacist. Increasingly in the 21st century, the role of the pharmacist is that of a healthcare professional, expert in medicines, and with a strong grounding in the science that underpins their practice. As such, the GPhC has adopted Miller’s Triangle (Figure 1) as the pedagogical model for the development of clinical competencies to ensure safe and effective practice, is established as early as possible within training programmes, including the undergraduate MPharm [3,4]. The introduction of this project in final year allows the student to progress from knows to does in Miller’s triangle (Figure 1).

Standard 10 of the GPhC’s 2011 education standards identifies the outcomes for the initial education and training of pharmacists [5]. An essential competency for the MPharm graduate is that they are expected to show how to ‘assess and critically evaluate evidence to support safe, rational and cost effective use of medicines’ (10.2.1b). They are also expected to be able to review the evidence base for current practice (10.2.1c). Several authors including Sackett (1979) [6] and Cochrane [7] advocated evidence-based practice (EBP) and called for “the conscientious use of current best evidence in making decisions about the care of individual patients or the delivery of health care services”. Archibald Cochrane defined EBP as “current best evidence is up-to-date information from relevant, valid research about the different forms of health care” [7].

The curriculum of the school of Pharmacy and Life sciences at Robert Gordon University (RGU) is designed so that evidence-based medicine underpins all teaching within the MPharm. During the early stages of the course, students demonstrate that they know how to use the evidence to review current practice in written examinations. This project allows students to use the evidence base to review current practice in a final (fourth) year SQF level 10 five-week research project in biomedical sciences.

Student research projects within pharmacy have always been problematical, issues of finance, technical support, and laboratory space negatively impact not only on staff availability but also on the scope of the student's project being relevant and meaningful to their future practice.

Increasing complexity in pharmaceutical intervention and advancement in person centered care mean that pharmacy students often find it difficult to make sense of the wealth of data available and to appreciate the importance of this to scientific evidence-based practice. During the first three years of the course students are introduced to, and develop a knowledge and understanding of, problem based learning and evidence based practice. However, by the time students undertake their final year project it is often the application of their knowledge that is in question rather than the knowledge itself. The critical awareness and analysis as to whether the evidence they are provided with is valid or not is often overlooked within a busy curricula.

The aims of this evaluation study were to explore the effectiveness of a novel practice-based approach to a biomedical science project, identify elements of difficulty in the process, and to explore students’ perceptions and reflections.

## 2. Methodology

This was a longitudinal qualitative case study research project conducted over four years with four or five students in each year. The research aim was to give a snapshot of the outcomes of the project to assess whether there was a need to adapt the project or the process. The project was split into three phases: mentoring, systematic review, and evaluation.

### 2.1. Phase 1: Mentoring

Students’ expectations of a biomedical science project is that all five weeks of data collection will be within a laboratory setting. Nevertheless, prior to the research project data collection period MPharm students involved in this type of project were asked to undertake a modified systematic review of a scientific topic following Cochrane guidelines [7] entitled “Global perspectives of pharmacovigilance and adverse drug reactions: a cytochrome P450 case study”.

During the third year of the MPharm, students undertake a narrative literature review in a related area to their fourth year project. Providing the opportunity to gather and interpret previous research in the area drawing a conclusion about the studies selected. Due to the students’ limited knowledge and appreciation of the area, it rapidly became apparent that they would require considerable assistance and mentoring when it came to undertaking a successful systematic review. Unlike a narrative review, systematic reviews contain explicit rules for selecting the studies to be included in the review. These rules guide the reviewers to which findings they can and cannot include in the review, thereby lessening the probability that bias will influence the conclusions. A structured format is used for consistent presentation of information and data [7].

On commencing this project, it was essential to manage their expectations of the research project and to mentor and support them on their systematic review journey. Central to the review was the scientific premise that in humans six of the 57 different cytochrome P450’s are the principal phase1 enzymes responsible for the metabolism of at least 60% of all drugs [8]. These P450s—namely CYP1A2, CYP2C9, CYP2C19, CYP2D6, CYP3A4, and CYP3A5—have polymorphic variants which affect their metabolic capacity [9]. As future pharmacists, it is important that the students are not only conversant with these particular enzymes but have the capacity to understand the importance of evidence based medicine relating to their role in drug metabolism.

Therefore, prior to the project start, weekly meetings were arranged to direct students to specific tasks building their knowledge, understanding, and confidence to successfully complete a systematic review. At the initial meeting, a student driven contract was instigated by which agreed milestones were set and achievements documented. Subsequent meetings provided the opportunity to identify challenges and concerns and to take action to resolve these in a timely manner, a brief synopsis of these is provided in Table 1.

### 2.2. The Systematic Review Process

The first step to developing a systematic review involves defining the purpose of the review: The most common method used when creating a question is to use the PICO method. PICO is an acronym which describes a framework around which a question can be built—the question should include a description of: the participants (the patient group); intervention (action being taken to improve patient’s health); comparison (a different intervention, to which the former intervention is being compared); and outcome (indicator of efficacy or harm resulting from intervention(s)). It should be noted that the comparison can in some cases be ‘no intervention’. PICO can on occasion be expanded to PICOS where ‘S’ is representative of the setting of the studies [7].

As one of the purposes of a systematic review is to identify relevant research, it is important that the search is as thorough as possible; all relevant keywords and the appropriate databases must be identified and recorded for optimum coverage of the body of evidence pertaining to the particular clinical subject. While this can be achieved by maximising the number of databases used, this will prolong the search time as more time is spent tailoring search strategies to the individual databases; in addition to the fact that one spends more time screening a larger volume of duplicated articles. In addition to being time-consuming, selecting a large quantity of databases to search has also been proven to have only a modest benefit over using a select few of the most appropriate databases [10]. For this reason, it was important to select databases which have an extensive coverage of the area of interest for the purpose of the review; the databases should not be too similar in their coverage, as this would cause duplication of effort with few or no unique papers yielded—essentially making the process less efficient. With this in mind, following initial meetings with the supervisor, students were required to seek the assistance of a librarian. Following which they were required to discuss their databases and reasoning with their supervisor. Search strategy development was an iterative process—it involved defining keywords and synonyms to search for, and how to combine them when searching; the strategy often needed to be refined and adjusted according to the database being searched, which was why piloting of the strategy was also a part of the initial development process. Individual sessions with the supervisor ensured students were cognisant not only with other search methods (inverted commas for phrase searching, truncation, and wildcards) specific to each database and the use of Boolean operators, but with Cochrane’s methodology and the necessity for the development and piloting of their search strategies [7].

After the search strategy had been developed and piloted, the next step was to assess results for relevance to the question. This involved confirming that inclusion and exclusion criteria were adequately defined, necessary to ensure only papers which specifically answered a student’s research question were included in the review. Five to six databases were selected for the final search which involved individual searches of each of the PICOS component, then a combined search of all the PICOS terms. In addition to electronic databases, other methods were employed to identify studies including snowballing and ‘grey literature’; literature which has not been peer-reviewed defined as “that which is produced on all levels of government, academics, business and industry in print and electronic formats, but which is not controlled by commercial publishers” [11]. At the end of the search process, all identified studies were uploaded to RefWorks, a reference management tool. The studies were assessed against the eligibility criteria in four stages:

- removing duplicates
- scoping assessment of titles
- assessing abstracts of the remaining studies
- reading of potentially eligible studies.

Once the relevant papers were identified they were analysed using data extraction tools. This was done systematically to improve validity of the review and extract necessary information about study characteristics and results from the included studies.

The data extraction proforma provided by the supervisor was initially developed from ones used by Mann et al. [12] and the North York Public Health Department, Ontario [13] amended to include statistical analysis. This was further adapted by the students to ensure relevance for their study, research question, inclusion/exclusion criteria, and articles. The students’ data extraction form was used for their studies to provide consistency within the review, improve validity and reliability, and reduce bias. The form was piloted and validated by extracting data from an initial study and checked by the supervisor prior to the commencement of further data extraction.

Finally, a protocol based on the critical appraisal skills assessment (CASP) tools [14] (which the students were familiar with from their third year of study) was decided for critical appraisal of the papers; the protocol was essential to ensure that the papers were being critiqued in a systematic way, reducing bias. This allowed the reviewer to make a valid conclusion based on the data found from the identified papers.

Although all projects were focused upon the generic “Global perspectives of pharmacovigilance and adverse drug reactions: a cytochrome P450 case study”, the titles were different and focused on such diverse topics as: an investigation into the adverse drug reactions and required pharmacokinetics associated with an increased dose of donepezil in Alzheimer's disease; impact of genetic polymorphisms in CYP2C9 on adverse drug reactions in patients administered with fluvastatin; gender specific responses to clopidigrel; comparison of P450 associated tardive dyskinesia in schizophrenics treated with atypical antipsychotics.

### 2.3. Evaluating the Process

After completion of this project the students were invited to take part in a ‘talking wall’ focus group on completion of the project. First described by Parsell, Gibbs, and Bligh in 1998 [15] to enhance interprofessional learning, ‘talking walls’ are a simple technique to collect data and enhance reflection by discussion with the other participants and was adapted from the commercial world. Our intention with this approach was to obtain student feedback to explore and analyse issues, opinions, and reflections and develop an action plan which could be fed forward to future years.

Student responses were analysed by grouping into themes and sub themes as summarised in Table 2 below.

## 3. Discussion

This use of systematic reviews as an alternative to a lab based project allowed the students to develop appropriate and relevant skills and enhanced knowledge of biomedical research methodology and the underpinning theory. This fits with the GPhC competencies required of an MPharm graduate [5]. This project introduced a robust understanding of evidence-based medicine to the students and by the end of the research they were very aware of the impact of guidelines and the background to their development. Their confidence and understanding of the topics grew during the review process and many of the students contacted regulators and guideline developers to discuss their topic area [2]. This was particularly impactful for several students whose initial perception of a drug class and their efficacy changed with the development of the evidence base during their systematic reviews. By the end of the project, it had been reinforced to all students that their knowledge of drugs had to be based on evidence rather than on assumption that ‘new’ meant ‘improved’ or ‘better’ for the patient.

## 4. Impact

The ‘sea change’ observed from the students was unexpected, however the positive change in attitude and the findings have been fed-forward to develop this approach further to subsequent biomedical science practice based projects. Particularly reassuring to the authors was the change in attitude from a predominantly negative to more positive. Using systematic reviews to develop knowledge and understanding of biomedical research and methodology has been perceived as successful by the students involved and enabled them to utilise this newly acquired skill on the remainder of their fourth year modules to great effect. Anecdotally, the majority of students indicated that these skills were important ones to take into their pharmacy practice—a serendipitous outcome of the project. This was a robust project that allowed the students to develop their own topic and gave them ownership of their own research. It empowered, engaged, and educated them in the development of their own evidence based practice.

For the school, the impact created a more effective use of laboratory space, and minimised the financial burden that science-based projects often bring with them. For the university, it utilised the skills and knowledge base of the support services, especially the library, in an effective and efficient manner. For academic staff within the school, it provided the opportunity for continuous professional development and the further integration of science and practice within the MPharm curriculum.

At the end of the project, students agreed that they could step outside their comfort zone and learn about biomedical topics in a novel evidence-based way.

## Figures and Tables

**Table 1:** Pre-project milestones and student tasks.

Week	Topic	Milestone	Student’s Task
1	Introduction	Research aim and objectives explained.	Identify paper for week 2 journal club (JC).
2	Journal club 1	Students presented at journal club. Discussion on evidence and critical appraisal tools.	Familiarise themselves with research area.
3	Research question	Initial discussion on research question Students provided with SIGN 50: a guideline developer's handbook	Decide area of interest (disease state) provide paper for next JC.
4	Journal club 2	Paper discussed, Methodology examined	Familiarise with bias.
5	Importance of bias	Understanding of research question; bias and confounding factors.	Identify a paper for week 6 JC.
6	Journal club	Development of search strategy	Develop search strategy for week 7.
7	Search strategy	Search strategy and plan how to undertake a systematic review.	Organise additional sessions on database searching and referencing with the librarian.

**Table 2:** Themes identified from the students responses in their own words.

Question	Student Response/Themes	−ve/+ve
What did you feel when you were first given your research topic?	Disappointed, a dawdle (Scottish for easy), lost, mixed feelings, worried/dread /discomfort, staff intimidating	Predominantly negative
How would you describe the experience of undertaking a systematic review?	Time consuming/ demanding, rewarding/ steep-learning-curve, lots-of-work, tedious, more work than lab projects, progress slow at times, liked more as went on, chose area, understanding beyond systematic approach, changing viewpoints, new and demanding, really useful.	Negative to positive
How do you feel now having completed your project?	Achievement, greater understanding of evidence base, relief, it was tough, happy, staff so nice, prerequisite knowledge, importance of research methods,	More positive than negative
What impact do you think the project will have on your future career?	Knowledge of evidence base, systematic approach, critically appraise, confidence to question therapeutic intervention, project—area of research	All +ve
